# The road ahead: emerging therapies for primary IgA nephropathy

**DOI:** 10.3389/fneph.2025.1545329

**Published:** 2025-02-04

**Authors:** Edward J. Filippone, Rakesh Gulati, John L. Farber

**Affiliations:** ^1^ Division of Nephrology, Department of Medicine, Sidney Kimmel Medical College at Thomas Jefferson University, Philadelphia, PA, United States; ^2^ Department of Pathology, Sidney Kimmel Medical College at Thomas Jefferson University, Philadelphia, PA, United States

**Keywords:** IgAN, sparsentan, iptacopan, corticosteroids, SGLT2 (sodium-glucose cotransporter 2) inhibitor, a proliferation inducing ligand (APRIL), B-cell activating factor (BAFF)

## Abstract

Primary IgA nephropathy (IgAN) is the most common form of primary glomerulopathy. A slowly progressive disease presenting in the young to middle-aged, most patients with reduced eGFR or proteinuria will progress to end-stage kidney disease (ESKD) in their lifetimes. The pathogenesis involves increased production of galactose-deficient IgA1 (Gd-IgA1) that forms immune complexes that deposit in the glomerulus, eliciting mesangial cell proliferation, inflammation, and complement activation. The backbone of therapy is supportive, including lifestyle modifications, strict blood pressure control, and renin-angiotensin system inhibition targeting proteinuria < 300 mg/day. Sodium-glucose transporter 2 inhibitors are indicated for persisting proteinuria or declining eGFR. Sparsentan is indicated for persisting proteinuria. Immunosuppression should be considered for all patients at risk for progression (persisting proteinuria and/or declining eGFR). To reduce Gd-IgA1 production, targeted-release budesonide is approved. Agents targeting B cell survival factors APRIL or BAFF/APRIL have significantly reduced Gd-IgA1 production and proteinuria in phase 2 trials but await phase 3 data for approval. To reduce inflammation, high-dose steroids are ineffective and toxic in Caucasian patients, although lower-dose regimens may be effective in Chinese patients. Complement inhibition is being actively studied. The factor B inhibitor iptacopan has conditional approval. The terminal pathway inhibitors cemdisiran and ravulizumab show promise in phase 2 studies. Our current approach for those requiring immunosuppression involves combining the reduction of Gd-IgA1 (nefecon) with suppressing the effects of inflammation (iptacopan). The optimal duration of such therapy is uncertain. Clearly, there is more to be learned with many trials underway.

## Introduction

Primary IgA nephropathy (IgAN) remains the most common primary glomerulopathy worldwide with an incidence of at least 2.5/100,000 population (1). There are marked racial differences with a higher incidence in Asian populations versus Caucasian populations and a lower incidence in those of African descent. Initially thought to be benign, it is now clear that up to 50% of patients will progress to ESKD within 20 years, and unless the rate of decline in eGFR is < 1/year [herein all eGFR expressed as ml/min/1.73m (2)], most patients will reach ESKD in their lifetimes (2).

The pathogenesis of IgAN is incompletely understood but involves four sequential hits (3). Hit-1 is the increased production of IgA1 deficient in hinge-region galactose residues (galactose deficient IgA1 or Gd-IgA1), usually circulating as polymers. Hit-2 is the production of anti-Gd-IgA1 antibodies (mainly IgG, but also IgA1, typically targeting exposed N-acetylgalactosamine residues). Hit-3 is the formation of immune complexes (serum proteins, polymeric Gd-IgA1, and anti-glycan antibodies). Hit-4 is the mesangial deposition of these complexes, with resulting mesangial cell proliferation, inflammation, complement activation, and podocyte injury that eventuates in glomerulosclerosis and interstitial fibrosis. Since normal individuals have both circulating Gd-IgA1 and anti-glycan antibodies, the underlying genetic risk (4, 5) and epigenetic (6) and bacterial modifications (7) are also important. The questions of who to immunosuppress and how to immunosuppress patients with primary IgAN are being aggressively investigated, and along with supportive therapy, are the focus of this review.

The clinical presentation of IgAN is variable. Some cases present with recurrent bouts of macroscopic hematuria precipitated by mucosal infections, including upper respiratory (synpharyngitic nephritis) or gastrointestinal infections. Others have urinary sediment abnormalities (glomerular hematuria and proteinuria) with a slow decline in eGFR. Some patients have florid nephrotic syndrome with minimal change disease in association with their mesangial IgA deposits and should be immediately offered immunosuppression similar to adult minimal change disease. Occasionally, patients may have a rapidly progressive course with crescentic IgAN and should also be immediately immunosuppressed with steroids and cyclophosphamide. We will not be further discussing these two situations requiring immediate immunosuppression as per KDIGO 2021 (8).

Given the relatively slow progression of most cases of IgAN, trials assessing the therapeutic efficacy of various immunosuppressants or other therapies rely on established surrogate endpoints. Acceptable surrogates for therapeutic trials in IgAN include proteinuria reduction (9) and rate of decline of eGFR (10). Significant proteinuria reduction at 9 - 12 months allows for conditional FDA approval, with full approval contingent upon significant effects on the rate of eGFR decline, typically over a 2-year period.

## Supportive therapy

Irrespective of the use of immunosuppression, the backbone of therapy for all IgAN patients is supportive, including lifestyle modifications, exercise, salt restriction, smoking cessation, strict blood pressure control (<120/70 if proteinuric), and renin-angiotensin-aldosterone system (RAS) inhibition (RASi) if hypertensive or proteinuric. In support of such therapy, of the 271 IgAN patients who initial qualified and then completed 6 months of a supportive therapy run-in in the Supportive Versus Immunosuppressive Therapy for the Treatment of Progressive IgA Nephropathy trial (Stop-IgAN), 94 (35%) were excluded for responding with proteinuria reduction (11). Additional supportive therapy now includes sodium-glucose transporter 2 (SGLT2i) inhibition and endothelin-1 (ET-1) receptor antagonism (see **Table 1**) (12).

**Table 1:** Placebo-controlled studies of supportive therapy for IgAN beyond RAS inhibition.

Drug	Mechanism	Study	Number	Duration	1^0^ endpoint(s)	Results	Comments
Dapagliflozin	SGLT2i	DAPA-CKD (13)	270	2.1 years	≥ 50% decline in eGFR, ESKD, death (kidney or CV)	HR 0.29 (95% CI 0.12-0.73) versus placebo	eGFR decline 3.5 vs 4.7/year UAC reduced 26% vs placebo
Empagliflozin	SGLT2i	EMPA-KIDNEY (14)	817	2 years	≥ 40% decline in eGFR, ESKD, eGFR <10, death from kidney failure	HR 0.67 (95% CI 0.46-0.97) versus placebo	Total slope of eGFR 28% less (95% CI -44 to -13) versus placebo
Sparsentan	Dual ET_A_RA AIIType1RA	PROTECT (18, 19)	404	2 years	Proteinuria change at 36 weeks	41% better than placebo, 40% better at 2 years	eGFR slope 1.0 better from baseline to 110 weeks (NS)
Atrasentan	ET_A_RA	ALIGN (16)	340	132 weeks	Proteinuria change at 36 weeks	36.1% better than placebo	Interim analysis of first 270 patients
SC0062	ET_A_RA	Heerspink (22)	131	24 weeks	Proteinuria change at 12 weeks	-27.6%, -20.5%, -51.6% at 5, 10, and 20 mg doses	No change in eGFR No increased edema

AIIType1RA, angiotensin II type 1 receptor antagonist; ET_A_R, endothelin A receptor; CV, cardiovascular; HR, hazard ratio; NS, not statistically significant; SGLT2i, sodium glucose transporter 2 inhibitor; UAC, urine albumin/creatinine ratio.

The beneficial effects of SGLT2i in chronic kidney disease (CKD) have been well-established in trials dedicated to patients with CKD, with post-hoc analyses from two trials confirming the benefit specifically in IgAN patients (13, 14). A meta-analysis of these CKD trials combined with heart failure trials and cardiovascular event trials showed a 51% reduction of kidney disease progression (≥ 50% decline in eGFR, ESKD, or death from kidney disease) (12). SGLT2is are now the standard of care for CKD, including IgAN.

It has become increasingly clear that ET-1, via activation of the ET_A_ receptor (ET_A_R), contributes to the pathogenesis of IgAN and other proteinuric glomerulopathies (15). Activation of the ET_A_R, present on multiple cell types within the kidney, elicits vasoconstriction, apoptosis, inflammation, and fibrosis (15). ET_A_R antagonism has shown benefit in other kidney diseases. The ET_A_R antagonist atrocentan reduced doubling of the serum creatinine level, ESKD, or kidney death in 2,648 patients with type 2 diabetes and CKD by 35% (p = 0.0047) in the SONAR Trial (16). Another ET_A_R antagonist, zibotentan, significantly reduced albuminuria when combined with the SGLT2i dapagliflozin in a 12-week trial versus dapagliflozin alone in a study of 449 patients with various types of kidney disease (ZENITH-CKD) (17).

Sparsentan is a dual antagonist of the angiotensin II type 1 receptor and the ET_A_R approved for use in IgAN. In an interim analysis of the phase 3 double-blind PROTECT trial (18) that compared sparsentan to irbesartan in 404 IgAN patients, a between-group relative reduction of 41% in least squares mean percent change from baseline in 24-hour protein/creatinine ratio (UPCR) in favor of sparsentan (least squares mean ration 0.59, p < 0.0001) was shown that led to accelerated FDA approval for the treatment of IgAN. In the confirmatory 2-year analysis, the proteinuria reduction seen at 36 weeks was maintained at 110 weeks (-40%), and the rate of change of eGFR from week 6 to week 110 was significantly reduced (1.1 ml/min/1.73m^2^ less decline with sparsentan, p = 0.037), although the rate of decline from baseline to 110 weeks was not significant (1.0 ml/min/1.73m^2^ less decline with sparsentan, p = 0.058) (19). The composite of ≥ 40% reduction in eGFR, ESKD, or death was reduced by 30% (NS).

A 24-week interim analysis of 20 adult patients with IgAN enrolled in a basket study of atrasentan, a selectve ET_A_R antagonist, in various glomerulopathies showed a geometric mean percent reduction of 24-hour UPCR from baseline of 57.4% (20). The phase 3 ALIGN study enrolled 404 IgAN patients into a double-blind randomized controlled trial (RCT) of atrasentan 0.75 mg/day orally or placebo, including 340 in the main stratum and an additional 64 in a separate stratum of patients receiving SGLT2is. In an interim analysis of the first 270 patients in the main stratum who completed 36 weeks, the geometric mean reduction in 24-hour UPCR ratio was 36.1% greater with atrasentan (p < 0.001), with a similar reduction versus placebo in the SGLT2i stratum (37.4%) (21). Although fluid retention and anemia (presumably from hemodilution) were greater with atrasentan, there were no cases of heart failure or severe edema, and no patients required a transfusion or discontinued treatment.

SC0062, a selective ET_A_R antagonist, was studied in a phase 2 placebo-controlled double-blind RCT in 131 Chinese IgAN patients that showed a significant placebo-corrected reduction in 24-hour UPCR at all doses tested (-27.6%, -20.5%, and -38.1% at 5, 10, and 20 mg daily doses, respectively, at 12 weeks, and -22.4%, -30.9%, and -51.6% at 24 weeks, respectively) (22). There was no difference in eGFR and no increase in peripheral edema.

In our opinion, ET_A_R antagonism is indicated in patients with persisting proteinuria (> 300 mg/day) in addition to lifestyle modification, RASi, and SGLT2i. Data in support of an added proteinuria reduction benefit combining sparsentan with an SGLT2i were recently presented (23, 24).

## Immunosuppression in IgAN

The use of immunosuppression in IgAN requires a personalized approach based on the likelihood of progression. The major adverse prognostic risk factors (biomarkers) are reduced eGFR, proteinuria, hypertension, and unfavorable histology (MEST-C). Younger patients carry a high lifetime risk for ESKD if eGFR decreases by > 1/year. Any patient with declining eGFR (for no other reason) and/or persisting proteinuria (> 300 mg/day) should be strongly considered for immunosuppression. The aims of immunosuppression are two-fold. On the one hand is the reduction of the production of Gd-IgA1 and associated antibodies to reduce mesangial deposition of immune complexes. On the other hand is the reduction of inflammation and its consequences induced by the pathogenic immune complexes that have deposited within the glomerulus.

## Galactose-deficient IgA1

Central to the pathogenesis of IgAN is enhanced production and circulation of Gd-IgA1 along with anti-Gd-IgA1 antibodies (mainly IgG and to a lesser extent IgA1). Although present in healthy humans, the mean levels of both Gd-IgA1 and anti-GdIgA1 antibodies are higher in IgAN patients (25), albeit with much overlap. The major source of Gd-IgA1 appears to be plasma cells residing in mucosal-associated lymphoid tissue (MALT), including gut-associated lymphoid tissue (GALT) in the terminal ileum (Peyer’s patches) and nasopharyngeal-associated lymphoid tissue (NALT) (26). Miss-homed IgA+ plasma cells residing in extra-mucosal sites (i.e., bone marrow) may also contribute (27).

Multiple agents have been and are being tested with the aim of reducing Gd-IgA1 production from MALT or elsewhere (**Table 2**). These include B cell depleting agents (rituximab), anti-plasma cell agents (felzartamab), a targeted-release formulation (TRF) of budesonide, and inhibitors of a proliferation-inducing ligand (APRIL) alone or both B-cell activating factor (BAFF) and APRIL (*vide infra*).

**Table 2:** Published randomized controlled trials of agents targeting a reduction in Gd-IgA by B-lineage cells.

Drug	Mechanism	Study	Number	Duration	1^0^ endpoint(s)	Results	Comments
TRF-budesonide (nefecon)	Steroids directly targeting B-lineage cells in GALT	NEFIGAN (46) (phase 2b)	149	9 months	24-hour UPCR	Ratio: 0.74 versus placebo	Gd-IgA1 levels not reported
		NefIgArd (49) (phase 3)	364	2 years	eGFR decline	5.05* better versus placebo (p <0.0001)	Gd-IgA1 levels not reported. 27% reduction in 9-month UPCR** Mild steroid related side-effects
Sibeprenlimab	mAb against APRIL	ENVISION (37) (Phase 2)	155	12 months	Change in 12-month UPCR	2 mg: 47.2% 4 mg: 58.6% 8 mg: 62% Placebo: 20%	Change in eGFR: 2 mg: -2.7; 4 mg: 0.2 8 mg: -1.5 Placebo: -7.4 IgA and Gd-IgA1 reduced by 65%
Atacicept	Fusion protein against APRIL and BAFF	JANUS (40) (phase 2)	16	Up to 72 weeks	Safety	Acceptable safety	Gd-IgA1, IgA, IgG, IgM dose-dependent reductions
		ORIGIN (41) (phase 2b)	116	36 weeks	Change in UPCR at 24 weeks	25% placebo-corrected reduction	35% at week 36 eGFR 5.7* better Gd-IgA1 reduced by 60%
		ORIGIN-Open label extension (42)	113	96 weeks	Change in Gd-IgA1	-66%	Hematuria: -75% UPCR: -52% Well tolerated
Telitacicept	Fusion protein against APRIL and BAFF	Lv et al. (43) (phase 2)	44	24 weeks	Change in 24-hour proteinuria versus placebo	240 mg: -0.88 g/day (p = 0.013) 160 mg dose: -0.29 g/day (NS)	eGFR remained stable Total IgA reduced by 45% Generally well tolerated
Hydroxychloroquine	TLR7/9 inhibition	Liu (60)	60	6 months	Percentage change in proteinuria	-48.4% versus +10.0%	Data on change in Gd-IgA1 or total IgA not presented
Mycophenolate	B/T cell inhibition	MAIN (54)	170	3 years	1) Composite (2x creatinine, ESKD, renal or CV death) 2) ≥ 30% or ≥ 50%*** decline in eGFR,	1) HR 0.23 2) HR 0.23	Data on change in Gd-IgA1 or total IgA not presented

*ml/min/1.73m^2^.

^**^in the first 199 patients (48).

***≥ 30% if eGFR >60, ≥ 50% if eGFR < 60.

APRIL, A proliferation-inducing ligand; BAFF, B cell activating factor; GALT, gut-associated lymphoid tissue; Gd-IgA1, galactose-deficient IgA1; HR, hazard ratio; mAb, monoclonal antibody; TLR, toll-like receptor; TRF, targeted-release formulation; UPCR, urine protein/creatinine ratio.

In an open-label RCT in 34 predominantly Caucasian proteinuric IgAN patients, rituximab with the standard therapy was compared to the standard therapy alone and effected significant peripheral B cell depletion; however, there was no benefit of reducing proteinuria or changing eGFR compared to standard therapy alone (28). Furthermore, there was no reduction in total IgA, Gd-IgA1`, or Gd-IgA1 autoantibodies. These negative results suggest that the prime producers of the pathogenic antibodies are CD20- plasma cells unresponsive to rituximab or possibly B cells residing in the GALT that are less accessible by rituximab. Furthermore, the trial was relatively small. No randomized controlled trials assessing B cell depletion with ofatumumab or obinutuzumab in IgAN have been published or are actively recruiting. Plasma cell depletion with the anti-CD38 monoclonal antibody felzartamab is under investigation in a phase 2a study (NCT05065970) but the results are not available. The plasma cell apoptosis-inducing agent bortezomib was given to eight proteinuric IgAN patients and produced complete remissions in three at 1 year that were maintained. No larger studies or RCTs have been published on proteasome inhibitors (29).

Central to the pathogenesis of IgAN is class switch recombination (CSR) of naïve B cells to IgA-producing B cells and eventually IgA-producing plasma cells. This CSR can occur via either T cell-dependent or T cell-independent pathways, facilitated in either case by dendritic or other cell (myeloid, epithelial) release of the soluble factors BAFF and APRIL. There are three receptors for these cytokines, the first is the BAFF receptor, responsive only to BAFF and most important in the early maturation of B cells to mature naïve B cells. The other 2 receptors, the transmembrane activator and calcium-modulator and cyclophilin ligand interactor (TACI, expressed on mature B cells and plasma cells) and B cell maturation antigen (BCMA, expressed on plasma cells) are responsive to both BAFF and APRIL and are required for CSR (TACI), further B-lineage maturation, and plasma cell survival (BCMA).

The pathogenic roles of BAFF and especially APRIL in IgAN are supported by multiple lines of evidence, reviewed in detail elsewhere (30, 31). For example, the transgenic overexpression of BAFF in mice results in elevated serum IgA and mesangial IgA-containing immune complex deposition (32), including aberrantly glycosylated IgA (33). The inhibition of APRIL was shown to be effective in a ddY mouse IgAN model (34). Clinically, levels of both BAFF (35) and APRIL (36) are elevated in IgAN patients and correlate with disease severity (35, 36). Hence, agents targeting these cytokines have been actively investigated for treating IgAN.

Blisibimod is a selective BAFF inhibitor studied in patients with systemic lupus. Data from BRIGHT-SC (NCT02062684), a phase 1/2 study in IgAN patients, were presented but never published. A phase 3 trial, BRILLIANT-SC: A Study of the Efficacy and Safety of Blisibimod Administration in Subjects With IgA Nephropathy, was withdrawn by the sponsor. No data on the selective BAFF inhibitor belimumab, used and approved for lupus nephritis, have been published in IgAN. However, the inhibition of APRIL alone or dual BAFF and APRIL inhibition are being actively investigated in IgAN.

Sibeprenlimab is a humanized IgG2 monoclonal antibody that binds and selectively inhibits APRIL. A phase 2 double-blind placebo-controlled trial (ENVISION) involved 155 proteinuric IgAN patients randomized to monthly intravenous (iv) infusions of sibeprenlimab 2 mg, 4 mg, 8 mg, or placebo for 12 months. Reductions of 24-hour UPCRs of 47%, 58%, 62% and 20%, respectively were found at 12 months (37). The changes from baseline in eGFR were -2.7, 0.2, -1.5, and -7.41 ml/min/1.73m^2^, respectively. In the 4 and 8 mg groups, the mean serum levels of both Gd-IgA1 and total IgA were reduced by approximately 65%, IgG by 35%, and IgM by 75%. Adverse events were similar to the placebo group. A phase 3 trial is active (Visionary Study, NCT05248646).

Zigakibart is a humanized monoclonal IgG4 APRIL inhibitor with positive results in a phase 1/2a trial in IgAN patients that was published only in abstract form (38). Additional efficacy and safety data were recently presented (39). A phase 3 trial (The BEYOND Study, NCT05852938) is ongoing.

Atacicept is a human recombinant fusion protein of the Fc portion of human IgG1 and TACI administered weekly by subcutaneous injection that inhibits both BAFF and APRIL. In the phase 2a JANUS study, atacicept 25 mg, 75 mg, or placebo were administered to 16 total patients with resulting dose-dependent reductions in Gd-IgA1, IgA, IgG, and IgM at 24 weeks that were sustained to week 72 (40). The larger phase 2b ORIGIN study was a randomized double-blind placebo-controlled trial of 116 mainly Caucasian and Asian patients with proteinuric IgAN administered atacicept 25 mg, 75 mg, 150 mg, or placebo weekly for up to 36 weeks. A placebo-corrected 25% reduction in 24-hour UPCR ratio, the primary endpoint, was found in the combined atacicept 75 mg/150 mg group; this reduction increased to 35% at 36 weeks (41). Additionally, Gd-IgA1 levels were reduced by over 60% and eGFR was stabilized (5.7 better versus placebo at 36 weeks).

A 60-week open-label extension of the ORIGIN trial enrolled 111 of the original 116 patients to receive 150 mg/week of atacicept for a total of 96 weeks of active therapy (60 weeks for those originally on placebo) and found a sustained 66% reduction in Gd-IgA1, a 75% reduction in the number of patients with baseline hematuria, and a 52% reduction in 24-hour UPCR; the eGFR declined at -0.6 ml/min/173m^2^/year (42). No safety issues emerged. A phase 3 trial, Atacicept in Subjects with IgA Nephropathy (ORIGIN 3, NCT04716231), is ongoing.

A similar IgG1-Fc-TACI fusion protein that inhibits both BAFF and APRIL, telitacicept, was studied in a phase 2 placebo-controlled trial. In total, 44 proteinuric Chinese IgAN patients were given weekly subcutaneous telitacicept 160 mg, 240 mg, or placebo for 24 weeks (43). The 240 mg dose reduced proteinuria by 49% (mean difference versus placebo 0.88, p = 0.013), although the reduction with 160 mg was non-significant. A phase 3 trial in Chinese patients is active (NCT05799287) and another is planned (NCT06654596). A third such dual BAFF/APRIL inhibitor, povetacicept, is under investigation in a trial restricted to IgAN patients (NCT06564142) and in phase 1b/2a trial patients with autoimmune kidney diseases, including IgAN (NCT05732402); very favorable results from NCT05732402 were recently presented (44).

It remains unclear whether targeting APRIL alone or BAFF plus APRIL would be optimal for reducing Gd-IA1 and associated antibodies in treating IgAN. Effectiveness and safety appear comparable based on available phase 2 trials. APRIL is critical given its role in the later stages of B cell maturation and as a plasma cell survival factor, with BAFF required for earlier maturation. Hence, dual inhibition may be more immunosuppressive but may have greater potential for infectious complications. Sibeprenlimab did not abrogate adequate Covid-19 vaccine IgG responses with all evaluable subjects responding to an mRNA vaccine in ENVISION (45). The rates of anti-Covid-19 antibody decline were similar to the placebo group. Efficacy and safety data from ongoing phase 3 trials should clarify this further. In our opinion, targeting APRIL (alone or with BAFF) will soon become a mainstay in the immunosuppressive treatment of IgAN.

In order to directly target B cells and plasma cells residing in the GALT with the intent to directly inhibit Gd-IgA1 production, targeted-release-formulation (TRF) budesonide (nefecon) was developed. A phase 2b double-blind RCT (NEFIGAN) of 149 patients showed that nefecon at either 16 mg/day (48 patients) or 8 mg/day (51 patients) significantly reduced urine protein/creatinine ratio at 9 months by 27.3% and 21.5%, respectively, compared to an increase of 2.7% with placebo (46). In an additional analysis of the NEFIGAN study, nefecon was shown to significantly reduce the serum levels of BAFF, IgA1, Gd-IgA1, and IgA-IgG immune complexes with the 16 mg dose (47).

NefIgArd was a phase 3 placebo-controlled double-blind RCT of nefecon 16 mg/day for 9 months in 364 IgAN patients with a 2-year follow-up (48, 49). In part A of the trial involving the first 199 patients, 9 months of nefecon reduced 24-hour urine protein/creatinine ratio by 31% versus placebo at 9 months (P = 0.0005), by 54% at 12 months (p < 0.0001), and significantly preserved eGFR (3.87 better with nefecon at 9 months that was maintained by 12 months) (48). Analysis of all 364 patients at the 2-year completion of the trial showed a significant reduction in the 2-year decline in eGFR, the primary end-point, by 5.05 versus placebo (P < 0.0001), a result that represented a 50% lower loss of kidney function over 2 years (49). The time to the composite of a ≥ 30% decrease in eGFR or ESKD was significantly reduced (hazard ratio 0.45, p = 0.0014). Time-averaged proteinuria between 12 and 24 months was reduced by 40.9% versus placebo (p < 0.0001), although more so at 12 months (49.7%) than at 24 months (30.1%). Steroid-related side effects were generally mild to moderate in severity, including edema, acne, muscle spasms, and hypertension. Based on available data, nefecon would be our first choice to potentially reduce Gd-IgA1 production in any patient warranting immunosuppression, with the main limitation being cost. Given the apparent waning effect on proteinuria from 12 to 24 months, the cyclical use of nefecon should be considered.

Mycophenolate mofetil (MMF) inhibits lymphocyte proliferation and antibody production and is a first line treatment for systemic lupus. A RCT in 40 Chinese IgAN patients revealed a significant reduction in total serum IgA levels and reduced binding of polymeric IgA to cultured mesangial cells with MMF, although Gd-IgA1 was not specifically assessed (50). Multiple observational studies and RCTs have evaluated the effectiveness of MMF in IgAN. Multiple meta-analyses found no benefit (51–53). More recently, Hou et al. randomized 170 Chinese patients to standard-of-care plus MMF versus standard-of-care alone (the MAIN trial) and found a significant 77% reduction in both the primary endpoint (doubling of serum creatinine, ESKD, or kidney or cardiovascular death) and progression of kidney disease (> 30 - 50% eGFR decline) (54). Data on the reduction in Gd-IgA1 or total IgA levels were not provided. A meta-analysis including the Hou et al. data confirmed the benefit of MMF in Chinese IgAN patients separately, but not in Caucasian patients (55).

The mucosal innate immune system is intricately associated with aberrant IgA1 glycosylation. Toll-like receptor (TLR) 9 binds to microbial DNA, enhancing IL-6 production which then downregulates the enzyme and co-enzyme involved in IgA1 O-glycosylation (56). Similarly, TLR7 can bind microbial RNA, resulting in increased Gd-IgA1 production (57). Hydroxychloroquine inhibits T cell-independent TLR7/9 activation, resulting in the downregulation of BAFF and APRIL production. Hydroxychloroquine has other immunosuppressive effects as well, including T cell inhibition. It is now the standard of care for preventing and treating lupus nephritis. Observational data and retrospective studies in Chinese IgAN patients support its use in this ethnic group (58, 59). Hydroxychloroquine significantly reduced proteinuria at 6 months in a placebo-controlled RCT involving 60 Chinese IgAN patients (60). Data showing effectiveness in Caucasian patients are currently not available.

Of the potential disease-modifying agents capable of reducing Gd-IgA1 production (the driving force of IgAN), nefecon is the only FDA-approved option for IgAN, limited mainly by cost but also by some steroid-related side effects. It remains our first choice of available and approved agents. Conclusive data on APRIL and dual APRIL/BAFF inhibition from phase 3 trials are awaited. MMF may be a consideration if IgAN is progressing and no other therapy is available, tolerable, or affordable, especially if the patient is Chinese. Hydroxychloroquine also showed promise in Chinese patients but requires more study before any general recommendations can be made.

## Targeting inflammation

Although not always pathogenic (lanthanic IgA deposits), deposition of IgA1-containing circulating immune complexes in the mesangium of IgAN patients elicits mesangial cell activation and proliferation, recruitment of inflammatory cells, complement activation (predominantly by the alternate pathway but also the lectin pathway), and podocyte injury that eventuates in glomerulosclerosis and interstitial fibrosis. Immunosuppression targeting such inflammation may be non-specific (e.g., systemic corticosteroids) or more directed at the underlying pathophysiology, namely complement inhibition (**Table 3**).

**Table 3:** Randomized controlled trials on immunosuppression to reduce glomerular inflammation and its consequences.

Drug*	Mechanism	Study	Number	Duration	1^0^ endpoint	Results	Comments
Corticosteroids**	Multiple	STOP-IgAN (11, 78)	109	3 years	Full remission eGFR decrease ≥ 15	OR 5.31 (p=0.02) OR 0.64 (p=0.32)	Control group supportive care only Combining all 162 pts: Full remission: OR 4.82 (p=0.01) eGFR decrease > 15: OR: 0.89 (p=0.75) No difference in annual eGFR decline More severe infections
Corticosteroids plus C/A***			53	3 years	Full remission eGFR decrease > 15	NS (p=0.30) OR 1.67 (p=0.42)	
Corticosteroids +/- C/A****	Multiple	STOPG-IgAN follow-up (62)	149	7.4 years	Composite (≥ 40% eGFR decline, ESKD, death)	HR: 1.2 (p=NS)	No difference if annual eGFR decline, ESKD, or eGFR loss of ≥ 40%
Corticosteroids	Multiple	TESTING (63)	503	4.2	Composite (≥ 40% eGFR decline, ESKD, death from kidney failure	HR: 0.53 (< 0.001)	9/11 secondary endpoints significant. HR for reduced dose regimen 0.27Serious adverse events increased
Iptacopan	Factor B inhibition	Zhang (64)	112	6 months	24-hour UPCR at 3 months	23% reduction	Further proteinuria reduction at 6 months Complement suppression: Bb, serum Wieslab, urinary C5b-9
		APPLAUSE-IgAN (65)	443	9 months	Change in 24-hour UPCR	38% reduction	Data restricted to first 250 patients to complete 9 months No increased infections
Cemdisiran	siRNA C5 suppressor	Barratt (70)	31	36 weeks	Change in 24-hour UPCR	37.4% reduction	C5 reduced 98% No safety issues
Ravulizumab	C5 inhibition	Lafayette (74)	66	26 weeks	Change in 24-hour proteinuria in g/day	30% reduction versus placebo	Effects persisted on open-label extension

*limited to recent multicenter randomized trials in patients on a background of renin-angiotensin system inhibition.

**eGFR ≥ 60 ml/min/1.73 m^2^.

***eGFR < 60 ml/min/1.73 m^2^.

****a retrospective follow-up up to 10 years of the original STOP-IgAN randomized trial.

C/A, cyclophosphamide followed by azathioprine.

The use of higher-dose systemic corticosteroids remains controversial, due mainly to the high incidence of side effects, especially infections, and variable efficacy. Earlier trials were of low quality and small size (61). Two more recent multicenter RCTs of steroids on a background of RASi concurred on the increased incidence of infections, but they were discordant in terms of efficacy. The STOP-IgAN trial in 309 predominantly Caucasian patients found that steroids alone or combined with cyclophosphamide/azathioprine (if eGFR < 60) were not more effective than supportive care in reducing the progression of kidney disease (11), even at long-term follow-up (62). In contrast, the Therapeutic Effects of Steroids in IgA Nephropathy Global Trial (TESTING) in 503 predominantly Asian patients found a significant 47% reduction in the primary end-point (eGFR decline ≥ 40%, ESKD, or death from kidney failure) and a significantly slower rate of eGFR decline (2.46/year) (63). We do not currently recommend high-dose steroids to suppress inflammation in IgAN, at least in Caucasian patients.

More exciting is the potential of complement inhibition that can target the alternate pathway, the lectin pathway, or the common pathway. A two-part double-blind placebo-controlled phase 2 dose-finding study of the alternate pathway oral Factor B inhibitor iptacopan (10, 50, 100, or 200 mg bid) included 112 total patients. A dose-response relationship was found with a significant 23% reduction in the 24-hour UPCR ratio at 3 months with the 200 mg bid dose. A further reduction was seen at 6 months with the 200 mg dose (64). Evidence of significant alternate pathway inhibition included reductions in plasma Bb levels, serum *ex vivo* alternate pathway activity (Wieslab assay), and urinary C5b-9 levels. The FDA granted accelerated approval for iptacopan in December 2023 (https://www.fda.gov/drugs/novel-drug-approvals-fda/novel-drug-approvals-2023) pending results of the full 2-year trial.

A phase 3 trial of iptacopan in 443 IgAN patients, A Multi-Center, Randomized, Double-Blind, Placebo-Controlled, Parallel Group Phase III Study to Evaluate the Efficacy and Safety of LNP023 in Primary IgA Nepthropathy (APPLAUSE-IgAN), is ongoing. An interim analysis of the first 250 patents to complete (or drop out by) 9 months was recently published (65). At nine months, iptacopan at 200 mg bid significantly reduced the mean 24-hour UPCR by 38.3% (p < 0.001) as compared to placebo, with a reduction in markedly elevated C5b-9 baseline levels to a range observed in normal individuals. There were no adverse safety signals. The effect of iptacopan on the decline of eGFR awaits completion of the trial.

IONIS-FB-LRx is an antisense inhibitor of Factor B that is administered by subcutaneous injection. In a study in IgAN patients, IONIS-FB-LRx reduced urinary protein by 44% at week 29 (company press release, Ionis Pharmaceuticals) in an unpublished phase 2 study. A phase 3 study is ongoing (NCT04014335). Two oral Factor D inhibitors, pelecopan and vemircopan, are under investigation with no results available.

Since activation of the terminal complement pathway at C5 has been shown to correlate with disease activity and progression of IgAN (66–68), multiple agents have been studied. C3 inhibition reduces the activity of all 3 C5 convertases and therefore depletes terminal pathway activity. Pegcetacoplan is a small peptide inhibitor of C3 that blocks its cleavage, thereby reducing terminal pathway activity. It is approved for use in the complement-mediated disease paroxysmal nocturnal hemoglobinuria. Dixon et al. administered pegcetacoplan as a daily infusion for 24 weeks with continuation twice weekly for another 24 weeks in a phase 2 study in 21 patients with complement-mediated glomerulopathies, including 6 with IgAN (69). Proteinuria reduction (24-hour urine protein/creatinine ratio), the primary end-point, was reduced from 1.3 to 1.2 mg/mg at 48 weeks in the IgAN patients.

Multiple agents targeting C5 have been studied in IgAN patients. In a phase 2 placebo-controlled 36-week trial of 31 primary IgAN patients, Barratt et al. studied cemdisiran, a subcutaneous, small-interfering RNA that inhibits C5 production. They showed a placebo-adjusted geometric mean change of -37.4% in 24-hour urine protein/creatine ratio (70). There was a slower decline in eGFR, reduced dipstick-grade hematuria, and a serum C5 reduction of 98.7% along with a 48% reduction of the alternate pathway and a 77% reduction of the classic pathway as assessed by Wieslab enzyme-linked immunosorbent assays (71).

The humanized, monoclonal C5 inhibitor eculizumab was reported to have a beneficial effect in 2 pediatric rapidly progressive cases of IgAN (one IgA vasculitis) (72, 73). We are unaware of any phase 2 or 3 randomized controlled trials of eculizumab in primary IgAN.

In a phase 2 double-blind placebo-controlled 26-week RCT, Lafayette et al. administered ravulizumab, a longer-acting second-generation humanized monoclonal C5 inhibitor, intravenously every 8 weeks to 43 patients versus placebo in 23 patients for 6 months (74). The primary end-point was change in proteinuria from baseline to week 26 and was significantly positive, -41.9% for ravulizumab versus -16.8% for placebo, giving a placebo-corrected treatment effect of 30.1% (P = 0.005). In an extension phase from weeks 26 to 50, all patients received open-label ravilizumab. At week 50, there was a 45% reduction in proteinuria from baseline in those originally randomized to ravulizumab and a 45% reduction in those crossing over from placebo. At week 50, eGFR declined by -3.9 with raviulizumab compared to -6.3 in those randomized to placebo. Ravulizumab was well tolerated. A phase 3 trial in 450 projected IgAN patients is ongoing (NCT06291376).

The C5a receptor inhibitor avacopan, shown to be effective and approved for use in ANCA-positive systemic vasculitis, was studied in a small, open-label pilot study in IgAN (75). Seven IgAN patients were given avacopan 30 mg twice daily for 12 weeks and had improvement in the slope of urine protein/creatinine ratio during the 12 weeks compared to the 8-week run-in period.

There is definite evidence of lectin pathway involvement, at least in some patients with IgAN, as shown by positive glomerular staining for C4d in the absence of C1q in perhaps a third of biopsies and glomerular deposition of mannan-binding lectin and ficolins. Narsoplimab, a monoclonal antibody targeting this pathway, was effective in a phase 2 trial (76), but the phase 3 trial was terminated by the sponsor due to a lack of efficacy.

## Discussion

Initially considered a relatively benign disease, it is now clear that most patients with IgAN are likely to reach ESKD in their lifetimes. Some progress more quickly than others, such that 20% to 50% are likely to reach ESKD by 20 years. The two biggest risk factors are declining eGFR and persisting proteinuria, compounded by hypertension and unfavorable histology. Persisting microscopic hematuria may signify ongoing inflammation. However, spontaneous remissions can occur. Han et al. followed 233 Korean IgAN patients of which 24 patients had nephrotic syndrome (77). Five of 12 non-immunosuppressed nephrotic patients had complete durable spontaneous remissions, including two with reduced eGFRs (< 45) and advanced pathology. We feel such a benign course is rare and its possibility should not dissuade therapy otherwise indicated.

All patients with IgAN deserve maximal supportive therapy as outlined above (**Table 4**). In our opinion, the question has shifted from *who* to immunosuppress to *who not* to immunosuppress. The approach to each patient should be individualized with the patient clearly involved in decision making. The underlying pathophysiology is probably not transitory but likely is lifelong. Initial immunosuppression should be aimed at reducing Gd-IgA1 (Hit 1), the driving force for progression. The currently approved therapy is nefecon for targeting the GALT, the main likely source of these antibodies. We await phase 3 efficacy and safety data on agents inhibiting APRIL or dual APRIL/BAFF, given the role of these cytokines in CSR to IgA production and the ability of their inhibitors to reduce Gd-IgA1 levels, reduce proteinuria, and preserve eGFR. We suspect inhibition of APRIL alone or APRIL plus BAFF will become a mainstay of therapy. Currently, MMF and/or hydroxychloroquine deserve consideration in Chinese patients if nefecon is not an option.

**Table 4:** Current recommendations 2025.

**Background therapy for all** Lifestyle modifications (smoking cessation, weight loss if overweight/obese, exercise) Strict blood pressure (BP) control (130/80 for all, <120/70 if proteinuric*) Renin-angiotensin system inhibition (RASi)** for BP control of if proteinuric Sodium glucose transporter 2 inhibitors (SGLT2i) if proteinuric or eGFR reduced (< 60) Sparsentan if proteinuric despite RASi and SGLT2i Potential, awaiting data: mineralocorticoid receptor antagonism (finerenone)
**Reduction in Gd-IgA1 production (immunosuppression)** Nefecon (FDA approved) Mycophenolate (off-label, only if Chinese, data in Caucasian patients not supportive) Hydroxychloroquine (off-label, only if Chinese, data in Caucasian patients unavailable) APRIL or dual APRIL/BAFF inhibition. Promising phase 2 studies. Await phase 3 data and FDA approval
**Suppression of inflammation and its effects (immunosuppression**) Corticosteroids (high infectious risk, not if Caucasian. Consider in Chinese with lower dose protocol of the TESTING trial). Alternate pathway inhibition: Iptacopan (conditional FDA approval) C5 inhibition (cemdisiran, ravulizumab, avacopan) awaiting phase 3 studies and FDA approval
**Major unanswered questions regarding immunosuppression** Should all patients with persisting proteinuria after background therapy be immunosuppressed? Should non-proteinuric patients with documented declining eGFR that is still > 60 be immunosuppressed? What role if any should persisting hematuria have in the decision to immunosuppress? Should nefecon*** be combined with iptacopan**** at the outset of therapy or should they be sequential? What is the optimal duration of initial therapy? Should repeat course(s) be considered? Should agents be alternated?

*defined as > 300 mg/day or > 300 mg/g of creatinine.

**angiotensin converting enzyme inhibitor or angiotensin receptor blocker.

***or other agents to reduce Gd-IgA1 (APRIL or APRIL/BAFF inhibitors) if approved.

****or other complement inhibitors if approved.

Concurrently with a reduction in Gd-IgA1 production, efforts to reduce glomerular inflammation and its effects should be considered. Steroids come with high toxicity with little or no benefit, at least in Caucasian patients. We do not recommend their use in Caucasian patients. The lower dose regimen used in the TESTING trial may be considered for Chinese patients. The reasons for such a differential response to steroids and to hydroxychloroquine and MMF noted above, are unclear. We suspect a genetic basis given the racial differences in prevalence and severity.

Complement inhibition appears to be a safer and more effective way to suppress inflammation in IgAN. The oral Factor B inhibitor iptacopan has received conditional FDA approval pending phase 3 trial results. We recommend its use based on efficacy in proteinuria reduction and safety. Inhibition of the terminal complement pathway appears promising as well with ravulizumab, cemdisiran, and possibly avacopan as candidates. Terminal complement inhibition would intuitively seem most likely to be beneficial in cases demonstrating thrombotic microangiopathy on biopsy, given the known benefit of eculizumab in atypical HUS. However, it may still have a role in cases lacking these findings.

At this time, combinational therapy with approved agents aimed at both pathogenic antibody reduction (nefecon) and complement inhibition (iptacopan) appears optimal, but it has not been studied in terms of additive benefit and safety. Lifelong therapy in some form will probably be needed in many if not most cases. Options include initial combinational therapy followed by sequential therapy or sequential therapy from the outset. In our opinion, young-to-middle-aged patients with ongoing proteinuria and hematuria, declining eGFR, and unfavorable histology deserve such initial combinational therapy, ideally with nefecon and iptacopan. Clearly, a personalized approach is optimal considering the risks of progression balanced by potential side-effects, ideally with strong input from an informed patient. Despite remarkable progress, much more needs to be learned.
